# Clinical and Molecular Relationships between COVID-19 and Feline Infectious Peritonitis (FIP)

**DOI:** 10.3390/v14030481

**Published:** 2022-02-26

**Authors:** Arjun N. Sweet, Nicole M. André, Alison E. Stout, Beth N. Licitra, Gary R. Whittaker

**Affiliations:** 1 Department of Microbiology & Immunology and Feline Health Center, College of Veterinary Medicine, Cornell University, Ithaca, NY 14853, USA; ans249@cornell.edu (A.N.S.); nma39@cornell.edu (N.M.A.); aek68@cornell.edu (A.E.S.); 2 Division of Nutritional Sciences, College of Human Ecology, Cornell University, Ithaca, NY 14853, USA

**Keywords:** feline infectious peritonitis, SARS-CoV-2, COVID-19, cats

## Abstract

The emergence of severe acute respiratory syndrome 2 (SARS-CoV-2) has led the medical and scientific community to address questions surrounding the pathogenesis and clinical presentation of COVID-19; however, relevant clinical models outside of humans are still lacking. In felines, a ubiquitous coronavirus, described as feline coronavirus (FCoV), can present as feline infectious peritonitis (FIP)—a leading cause of mortality in young cats that is characterized as a severe, systemic inflammation. The diverse extrapulmonary signs of FIP and rapidly progressive disease course, coupled with a closely related etiologic agent, present a degree of overlap with COVID-19. This paper will explore the molecular and clinical relationships between FIP and COVID-19. While key differences between the two syndromes exist, these similarities support further examination of feline coronaviruses as a naturally occurring clinical model for coronavirus disease in humans.

## 1. Introduction

In the 1960s, feline infectious peritonitis (FIP) was described as a disease in domestic cats and later recognized to be of a viral etiology, specifically feline coronavirus (FCoV) [1,2]. In a majority of cats, infection with FCoV results in mild to inapparent clinical signs; however, a small subset will develop severe illness and succumb to the systemic form of the disease, known as FIP [3]. In the years since its discovery, many features of FCoV have remained elusive. Likewise, the COVID-19 pandemic, caused by the emergence of SARS-CoV-2, has posed many equally challenging questions regarding epidemiology pathogenesis, transmissibility, and treatment. The widespread transmission of FCoV/SARS-CoV-2 and the insidious onset of severe signs for both FIP and COVID-19 limits the ability for early disease detection—what may begin as only mild or even inapparent clinical signs or symptoms can rapidly lead to systemic disease [3,4]. We believe that FIP may represent a valuable, naturally occurring extrapulmonary model of COVID-19.

**Figure 1:**
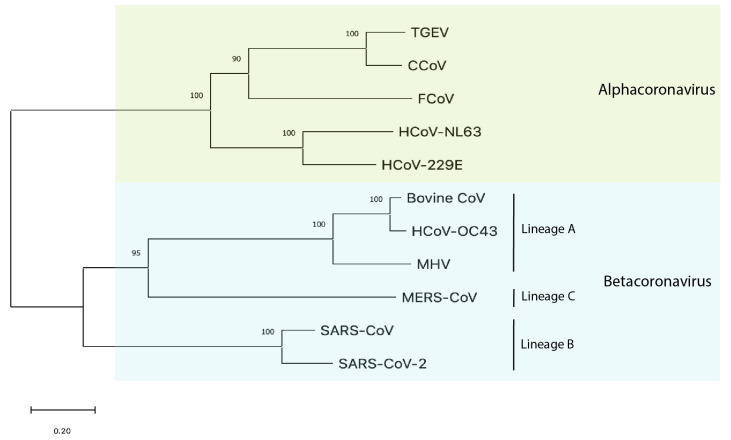
Phylogenetic tree of the spike proteins of selected coronaviruses. A maximum likelihood phylogenetic tree was constructed using MEGAX (100 bootstraps) from a multiple sequence alignment of spike protein sequences. Spike amino acid sequences were obtained from NCBI GenBank. Accession numbers are: transmissible gastroenteritis virus/TGEV (P07946), severe acute respiratory syndrome coronavirus 2/SARS-CoV-2 (YP_009724390.1), Middle East respiratory syndrome coronavirus/MERS-CoV(AFS88936.1), mouse hepatitis virus/MHV-1 (ACN89742), severe acute respiratory syndrome coronavirus/SARS-CoV (AAT74874.1), feline coronavirus/FCoV-Black (EU186072.1), bovine coronavirus/BCoV (P15777), canine coronavirus/CCoV (AY436637.1), human coronavirus/HCoV-OC43(NC_006213.1), HCoV-229E(NC_002645.1), and HCoV-229E(NC_002645.1).

Both FCoV and SARS-CoV-2 belong to the family *Coronaviridae* [4,5], albeit in distinct genera (Figure 1). FCoV along with similar animal coronaviruses, such as canine coronavirus (CCoV) and transmissible gastroenteritis virus (TGEV) in pigs, belong to the genus alphacoronavirus. Also grouped within the alphacoronavirus genus are the community-acquired respiratory (CAR) human coronaviruses 229E and NL63 [6] with the latter associated with the common cold, croup, and possibly Kawasaki disease in children [7]. In contrast, SARS-CoV-2 along with SARS-CoV (the cause of the 2002–2003 severe acute respiratory syndrome outbreak) and the Middle East respiratory syndrome coronavirus (MERS-CoV) belong to the genus betacoronavirus [8], with SARS-CoV-2 and SARS-CoV in lineage B (sarbecovirus) and MERS-CoV in lineage C (merbecovirus). The less closely related betacoronaviruses include the CAR human coronavirus OC43 (associated with the common cold), mouse hepatitis virus (MHV), and bovine coronavirus, which is associated with pneumonia and diarrhea in cattle; these viruses are in lineage A (embecovirus).

FCoV can be classified in two ways, the first being relevant to the disease form. Feline enteric coronavirus (FECV) is considered the causative agent of the mild gastrointestinal form of disease, while feline infectious peritonitis virus (FIPV) is associated with the lethal systemic infection known as FIP [3]. FIPV is distinguished from FECV by its ability to infect and replicate efficiently within monocytes and macrophages [9] triggering systemic inflammation. FIPV is associated with a spectrum of clinical outcomes. At one end of the spectrum is effusive, or ‘wet’ FIP, which is rapidly progressive and involves accumulation of a highly proteinaceous exudate in the abdominal and/or thoracic cavities. At the other end of the spectrum is non-effusive, or ‘dry’ FIP, which can affect many organ systems but is typically characterized by neurologic and ocular signs. Non-effusive FIP generally has a more protracted disease course and is less common than its effusive counterpart. FCoV can also be classified into two serotypes—type I or type II—based on major differences in the viral spike protein that affect receptor binding and antibody response [10]. The receptor for type II FCoV is feline aminopeptidase N (fAPN) [11], while the receptor for type I viruses is unidentified. Type I FCoV accounts for the vast majority of FIP cases [12].

SARS-CoV-2 classification into different variants based on genetic mutations is ongoing as the virus continues to evolve. Viral lineages demonstrating the potential for increased transmissibility, treatment resistance, vaccine escape, or increased morbidity and mortality have been designated variants of concern (VOC). The spectrum of disease associated with COVID-19 is broad and ranges from asymptomatic and mild infections to acute respiratory distress syndrome (ARDS), systemic inflammatory response syndrome (SIRS), and multiorgan failure and death. Systemic inflammation in SARS-CoV-2 is not linked to macrophage and monocytes (as in FIP), but it does account for a wide range of extrapulmonary signs. This appears to involve the SARS-CoV-2 receptor, angiotensin converting enzyme-2 (ACE-2), which plays an important role in the renin–angiotensin system and the development of a pro-inflammatory state [13]. Multisystemic inflammatoy syndrome (MIS) of children and adults, as well as post acute sequelea of SARS-CoV-2 infection (PASC), also known as ‘long COVID’, are potential outcomes of a COVID-19 infection.

## 2. Transmission

As a group, coronaviruses are known for their ability to cause both respiratory and enteric disease and are generally transmitted by one or both routes. While FCoV is considered fecal–oral and SARS-CoV-2 is primarily respiratory, COVID-19 patients can shed infectious virus in their stool [14], often for prolonged periods, and FCoV can readily infect via the oronasal route, a common method of challenge for experimental inoculation of cats [15].

In most cases, FCoV infection is self-limiting, and although the virus can be detected systemically, replication outside of the intestinal epithelium is poor. This form of the virus, termed FECV, is readily transmissible via the fecal–oral/oronasal routes, with the common sources of infection involving shared litter boxes and ingestion of viral particles through grooming. The current understanding of the development of FIP involves internal mutation: in a small subset of FECV cases, a complex combination of host and viral factors leads to mutation(s) allowing for efficient replication within macrophage and monocyte lineages [16]. These lethal variants are classified as FIPV and are associated with systemic inflammation, organ failure, and death. FIPV is generally considered to be nontransmissible as the factors that increase its tropism to macrophages appear to restrict it from fecal–oral spread [17]. Outbreaks of FIP have been reported in catteries and shelters. In these situations, the stress of overcrowding and high levels of virus in the environment may favor the transition of FECV into FIPV. There is evidence some strains of FCoV may be more predisposed to this transition than others [18,19].

A SARS-CoV-2 infection primarily targets the respiratory epithelium, but as with FCoV, the virus can be detected systemically without corresponding signs of infection [20,21]. Asymptomatic individuals are a well-documented source of SARS-CoV-2 [22,23,24], and transmission involves both inhalation of aerosols and contact with droplets [25]. Incubation periods for SARS-CoV-2 and FECV range from 2 to 14 days [26]. The incubation period of FIP is highly variable, influenced by time to internal mutation and the individual’s immune response. Onset of FIP may occur weeks to months after initial infection [27,28,29,30]. Multisystemic inflammatory syndrome in children (MIS-C), a serious manifestation of SARS-CoV-2, also lags behind initial infection with a median onset of 4 weeks. No viral factors have been associated with the development of MIS-C, but an immune mediated component is suspected.

Vertical transmission of FIP through the placenta or milk is thought to be uncommon. In an early experimental study where a lactating cat was infected, one of four kittens succumbed to FIP [28]. More often, maternal antibodies appear to be effective in preventing transmission up until about six weeks of age, at which time waning antibody levels make kittens susceptible to transmission via the fecal–oral route [31]. However, this maternally-derived immunity can be overcome at an early age by high levels of FCoV exposure—with a Swiss study demonstrating kittens in large catteries showing infection at two weeks of age [32,33]. Vertical transmission is a concern with a SARS-CoV-2 infection. Placental transmission is uncommon but has been documented in fetuses of SARS-CoV-2-infected mothers [34,35,36], evidenced by the detection of the virus in the amniotic fluid, neonatal blood, umbilical cord blood, and placental tissue. Transmission events have been documented both in early and late pregnancy, but neonatal infection with SARS-CoV-2 may not always occur in utero. Infection may also occur during delivery or close contact with the mother. Neonatal outcomes of COVID-19-infected mothers remain an area of study, with challenges in distinguishing between the impacts of a SARS-CoV-2 infection and maternal comorbidities. Nevertheless, infection of newborns does not appear to be without consequence, with one analysis noting approximately 50% of infected newborns demonstrating clinical features related to COVID-19, including fever and respiratory and gastrointestinal signs [37].

## 3. General Clinical Presentation

Clinical signs and symptoms associated with both FIP and COVID-19 include fever, diarrhea, depression, weakness, anorexia, and dyspnea [1]. The typical presentation of COVID-19 commonly involves nonspecific symptoms including fever, dry cough, fatigue, shortness of breath, and myalgia [38]. Anosmia (loss of smell) and ageusia (loss of taste) have also been frequently reported with COVID-19 and present as more specific symptomatic markers of the disease [39]. Pneumonia, acute respiratory distress syndrome (ARDS), and sepsis can be seen. Males seem to be at a higher risk of developing more severe manifestations of COVID-19 [40,41], with several small-scale studies supporting the same association between male sex and the development of FIP in cats [42,43].

**Figure 2:**
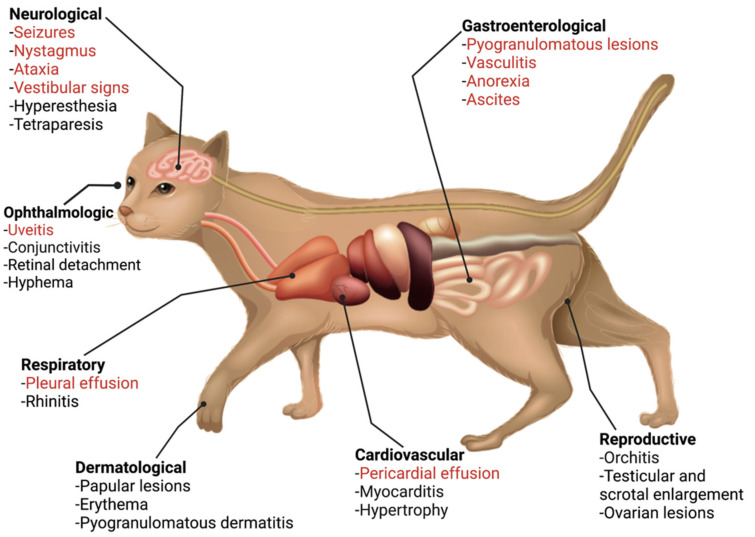
Summary of the systemic clinical signs and pathologies associated with FIP. FIP is well known to be a systemic infection with a diverse presentation. The possible systemic clinical signs associated with FIP, encompassing the organ systems that are also affected by COVID-19, are summarized. The most common signs of FIP are colored in red.

**Figure 3:**
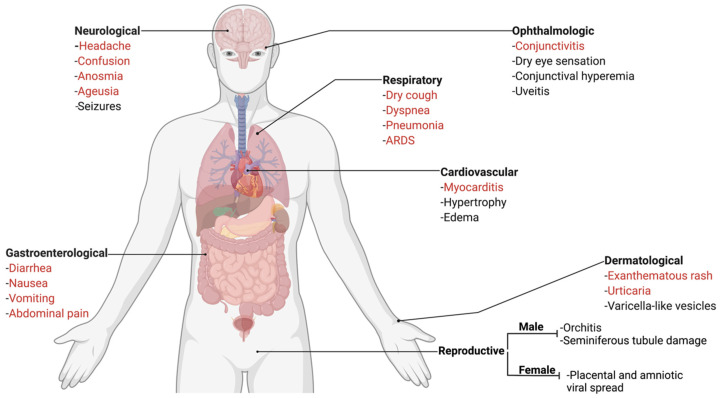
Summary of the systemic clinical signs, symptoms, and pathologies associated with COVID-19. The respiratory signs of COVID-19 are the main manifestation of the disease. However, a SARS-CoV-2 infection in humans can also result in diverse extrapulmonary signs. The systemic clinical signs and symptoms associated with COVID-19, encompassing the organ systems that are also affected by FIP, are summarized here. The most common signs of COVID-19 are colored in red. ARDS refers to acute respiratory distress syndrome.

The classic presentation of FIP is the development of effusion in the abdominal and/or thoracic cavity; while this presentation has been reported with COVID-19 [44], it is extremely rare. Beyond this, FIP presents in a range of body systems, which have similarity to the extrapulmonary presentations of COVID-19 (Figure 2 and Figure 3). The most similar feature between both diseases is endothelial dysfunction. Vasculitis is the hallmark of FIP pathology [45,46] with lesions characterized by perivascular edema and infiltration, degeneration of vascular wall, and endothelial proliferation [47]. In the case of COVID-19, it has been suggested that extrapulmonary signs are due to viral-mediated endotheliitis, leading to vasculitis, primarily in veins with little arteriolar involvement [48,49]. In the next sections, we describe these extrapulmonary signs and point out key similarities and differences.

## 4. Biomarkers

Inflammatory biomarkers are of interest as prognostic indicators in COVID-19 and as a means of differentiating FIP from other diseases. In FIP, IL-6 expression appears to be upregulated in the ascitic fluid of FIP-infected cats, possibly via increased expression in the heart and liver [50,51]. Other acute phase proteins are also upregulated in FIP infection. Alpha-1-acid glycoprotein (AGP) has been explored as a diagnostic marker of FIP, but it can be upregulated in other conditions, thereby limiting its specificity [52,53]. Serum amyloid A (SAA) is another acute phase protein that appears to distinguish between FIPV and FECV infection, with FIPV-infected cats demonstrating higher levels of SAA compared to FECV-infected cats and non-SPF controls [54], but it has limited use in differentiating FIP from other effusive conditions [55].

Similar to what has been documented for FCoV, individuals suffering from severe cases of COVID-19 have higher levels of SAA compared to those with a milder form of COVID-19 [56]. Higher levels of SAA are also reported in patients who died from COVID-19 as compared to survivors [57]. C-reactive protein (CRP) is another marker that shows promise as a biomarker in both FCoV and SARS-CoV-2 infections. CRP synthesis by the liver is induced by IL-6 expression as a response to inflammation [58] and is elevated in cases of FIP [59]. Elevated CRP levels in the early stages of COVID-19 have been associated with more severe disease and greater mortality [60,61,62], leading to recommendation for its use as a prognostic indicator when evaluating risk in patients hospitalized for COVID-19. In contrast, one meta-review found that IL-6 levels, while elevated, were at least one order of magnitude lower in COVID-19 patients than in those with non-COVID-19-related ARDS and sepsis, suggesting a different mechanism of immune dysregulation [63].

D-dimer, though not specific to COVID-19 or FIP, is another biomarker of interest. D-dimer is released from the breakdown of fibrin and is used as a clinical tool for ruling out thromboembolism [64]. Thrombotic events have been frequently documented in COVID-19 across multiple organ systems [65,66], and increased D-dimer levels are associated with greater morbidity and mortality [67,68]. Likewise, thrombotic events can occur in FIP, and high levels of D-dimers along with other signs of disseminate intravascular coagulation (DIC) can be seen in the end stages of FIP in both natural and experimental infections [69,70].

## 5. Pathophysiology

### 5.1. Neurological

FIP is one of the leading infectious neurological diseases in cats and the signs associated with central nervous system (CNS) infection are well documented [71]. CNS symptoms are recorded in about 40 percent of dry FIP cases and may appear as nystagmus, torticollis, ataxia, paralysis, altered behavior, altered mentation, and seizures [72]. The wide spectrum of signs supports the conclusion that infection is not limited to a specific portion of the CNS [73]. Infection within the CNS is limited to monocyte and macrophage lineages and results in pyogranulomatous and lymphoplasmacytic inflammation, which typically affects the leptomeninges, choroid plexus, and periventricular parenchyma [74].

Documentation of neurological signs associated with SARS-CoV-2 infection of the CNS is limited in comparison to other coronaviruses [75]. The observed signs are diverse, ranging from headache and confusion to seizures and acute cerebrovascular events [76]. The detection of the virus in the brain is uncommon, suggesting that signs may not directly linked to CNS infection. Viral particles have been observed in neural capillary endothelial cells and in a subset of cranial nerves, although such detection is not correlated with the severity of neurological signs [77]. Often, evidence of direct infection is not apparent. Instead, inflammatory mediators such as activated microglia are noted and may contribute to microvascular damage and disease. [78,79].

Further comparison of the neuroinflammatory properties of SARS-CoV-2 and FCoV may bring new perspective to the neurological manifestations of COVID-19. Further examination of neurologic signs associated with SARS-CoV-2 is vital for understanding the progression of COVID-19 and the extent of CNS infection.

### 5.2. Ophthalmological

Ocular manifestations of FIP are more prevalent in the dry form of the disease [80]. Mydriasis, iritis, retinal detachment, conjunctivitis, hyphema, and keratic precipitates have been observed [81]. The most common ocular manifestation of FIP is uveitis, which can affect both the anterior and posterior uvea [80]. The viral antigen can also be detected in the epithelial cells of the nictitating membrane, however the detection of the viral antigen does not distinguish between FECV and FIPV [82].

Ocular presentations of COVID-19 include conjunctivitis, chemosis, epiphora, conjunctival hyperemia, and increased tear production [83]. Uveitis—a common ocular presentation of FIP—has also been observed with a SARS-CoV-2 infection [84,85]. Viral detection in the tear fluid led to concern for ocular transmission in the early months of the COVID-19 pandemic [83,86]. SARS-CoV-2 RNA has been detected in lacrimal secretions and has been isolated from ocular secretions, supporting the potential of ophthalmologic transmission [87,88]. Interestingly, in the aforementioned case study in China, only 2 out of the 12 patients with ophthalmologic signs returned positive conjunctival tests, suggesting limited sensitivity in the detection of the virus from conjunctival samples [83].

### 5.3. Cardiovascular

Pericardial effusion is a less common manifestation of FIP but is well documented in the literature [26,89,90,91]. FCoV has been detected in the pericardium of a cat with recurrent pericardial effusion that later developed neurologic signs [92]. Direct FCoV infection of the heart was documented in a 2019 case study that reported FIP-associated myocarditis with notable left ventricular hypertrophy and atrial enlargement [93]. Immunohistochemistry (IHC) revealed the presence of FCoV-infected macrophages and associated pyogranulomatous lesions. [26]. Interestingly, a severe SARS-CoV-2 infection with evidence of viral replication within the heart and lungs was recently documented in a cat with pre-existing hypertrophic cardiomyopathy (HCM) [94].

In contrast to FIP, cardiac injury associated with a SARS-CoV-2 infection appears to be much more widespread. A 187-patient study found 27.8% of COVID-19 cases to have evidence of myocardial injury as evidenced by elevated cardiac troponin (TnT) levels [95]. High TnT levels were, in turn, associated with a higher mortality. A retrospective multicenter study of 68 COVID-19 noted 27 deaths that could be attributed to myocardial damage and/or circulatory failure as one of the primary causes of mortality, with elevated C-reactive protein and IL-6 levels linked to higher mortality [96]. The elevation of such inflammatory biomarkers in the blood suggests the rapid inflammatory nature of COVID-19 may have a particularly detrimental impact on cardiac function. Diffuse edema as well as increased wall thickness and hypokinesis have been noted in a COVID-19 infection [97]. Cardiac tamponade has also been observed in patients with COVID-19, with the pericardial fluid having detectable levels of SARS-CoV-2 [98]. In contrast to FIP, in which direct invasion of FCoV-infected macrophages in the myocardium has been observed in myocarditis, a SARS-CoV-2 infection of the myocardium is not clearly associated with mononuclear cell infiltration or myocarditis [99]. This leads to the consideration of more systemic factors in adverse cardiac outcomes—particularly the dysregulation of inflammatory cytokines. The impact of a SARS-CoV-2 infection on the cardiovascular system is an important element in our growing understanding of morbidity and mortality associated with COVID-19.

### 5.4. Gastroenterological

FCoV is shed in the feces and transmitted by the oronasal route. Initial FCoV infection is targeted to the intestinal tract—infection may be subclinical or cats may develop diarrhea, and less commonly, vomiting. Primary infection lasts several months, and the virus can be shed for months to years [100,101]. Colonic columnar epithelial cells appear to serve as a reservoir for persistent infection and shedding [21]. Signs tend to be mild and self-limiting, and only a small subset of animals will progress to FIP. Fibrinous serositis and pyogranulomatous lesions with vasculitis are the classic lesions of FIP and can be found in both the small and large intestines of affected cats [102]. FIP can cause solitary mass lesions in the intestinal wall, although this is considered an uncommon presentation (26/156 cats in one study) [103]. These tend to be located in the colon or ileocecal junction and are pyogranulomatous in nature.

Gastroenterological signs are widely reported with a COVID-19 infection. ACE2, the cellular receptor for SARS-CoV-2, is widely expressed in the glandular cells of gastric, duodenal, and rectal epithelium. Viral RNA and nucleocapsid have been detected in these tissues [104], supporting their suitability for SARS-CoV-2 replication. Gastrointestinal (GI) symptoms range from general lack of appetite to diarrhea, nausea, vomiting, and abdominal pain [105,106]. Excluding the less-specific symptom of a lack of appetite, several meta-analyses estimate the prevalence of GI symptoms in COVID-19 patients to be approximately 10% to 20%, with the most frequently reported symptom being diarrhea [106,107,108]. Interestingly, GI symptoms in COVID-19 have been observed without accompaniment of respiratory signs [105].

Viral shedding in feces has been of particular concern with COVID-19, as SARS-CoV-2 RNA can continue to be present in fecal matter even after reaching undetectable levels in upper respiratory samples [109]. While the detection of viral RNA in feces itself is not necessarily indicative of the presence of infectious virions, viable viral particles have been detected in feces [110]. The viral antigen has also persisted in the cells of the gastrointestinal tract in the convalescent phase, up to 6 months after recovery [20]. In one case study, persistent colonic infection was linked to persistent gastrointestinal signs in a case of ‘long COVID’ [111], introducing a parallel to the role of the colonic epithelium as a reservoir for FCoV.

### 5.5. Dermatology

Dermatological lesions have been reported in both SARS-CoV-2 and FIPV infections. Although rare, papular cutaneous lesions are the primary dermatologic manifestation of FIP, with the few available case reports documenting papules [81,112,113,114]. On histologic examination, pyogranulomatous dermatitis, phlebitis, periphlebitis, vasculitis, and necrosis were noted in several FIP case reports [81,112,113,114,115].

The first report of dermatological manifestations associated with COVID-19 was observed in Lecco Hospital in Lombardy, Italy [116]. In this study 18/88 patients (20.4%) exhibited cutaneous involvement where 8/18 were observed upon onset and 10/18 after hospitalization [116]. Clinical symptoms included erythematous rash (14/18 patients), diffuse urticaria (3/18 patients), and chickenpox-like vesicles (1/18 patients) [116]. The lesions were primarily observed on the trunk (torso) and pruritus was mild or absent [116]. The continuation of the pandemic has seen greater characterization of the first-observed dermatological signs as well as identification of more rare presentations. An exanthematous rash, often characterized by maculopapular lesions, appears to be the most common dermatological manifestation of COVID-19 [117,118]. Urticaria also appears to be another prevalent dermatological sign [118,119]. Importantly, neither exanthema nor urticaria are specific for COVID-19, limiting their positive predictive value. Varicella-like exanthema have been observed in a SARS-CoV-2 infection and may be a more specific presentation given its low prevalence in viral illness. Especially with a lack of oral lesions and pruritis observed in the COVID-19-associated rash, coupled with a previous history of varicella infection, the specificity of this presentation is strengthened [118].

### 5.6. Theriogenological

Orchitis and periorchitis have been observed in multiple cases of FIP with fibrinopurulent or granulomatous infiltrates as well as hypoplastic testes [1,26,120]. Inflammatory mediators from the tunics surrounding the testes have caused testicular enlargement in FIP cats [26,120]. In effusive FIP, scrotal enlargement has been observed due to the edema and peritonitis of tunics [16]. Despite the obvious pathology to the feline male reproductive system, FCoV was not detected in semen, decreasing the likelihood of a venereal route of transmission [121]. Female reproductive pathology of FIP is less documented in the literature but has been observed with macroscopic lesions present in the ovaries of FIPV-infected cats. The surrounding vessels of the uterus and ovaries in these cats were observed to be surrounded by lymphocytes, macrophages, plasma cells, and neutrophils [122].

Analogous to FIP, the pathology of COVID-19 also appears to be evident in the human male reproductive system. In one study, examining the testes of 12 COVID-19 patients, there was edema as well as lymphocytic and histiocytic infiltrate—consistent with viral orchitis [123]. These samples were also characterized by damage to the seminiferous tubules, with a notable impact on the Sertoli cells, as well as decreased numbers of Leydig cells. In a separate study, the damage to germ cells was more pronounced despite similar levels of the Sertoli cells between SARS-CoV-2-infected individuals and uninfected controls, posing a more direct link between infection and fertility [124]. The extent to which SARS-CoV-2 can persist in the male reproductive tract remains under investigation. While SARS-CoV-2 has been detected in human semen, whether this represents actual infection of the testicles or is a result of a compromised blood–epididymal/deferens barrier is questioned [125,126].

Our understanding of COVID-19 in the human female reproductive system remains limited by the amount of literature and the sample sizes of existing studies. Nevertheless, comprehending the extent of SARS-CoV-2 in the female reproductive tract is imperative in recognizing any deleterious impacts on fertility. ACE2 is expressed in the ovaries, oocytes, and uterus, but the limited co-expression of proteases such as TMPRSS2 and cathepsins L and B with ACE2 raises questions about the likelihood of ovarian/uterine infection [127,128]. While one study of 35 women diagnosed with COVID-19 returned no detection of SARS-CoV-2 in vaginal fluid or exfoliated cells from the cervix, SARS-CoV-2 was detected in vaginal fluid via RT-PCR in a case study from Italy (Ct 37.2 at day 7 and Ct 32.9 at day 20 from symptom onset), suggesting that infection of the female reproductive system may be possible [129,130].

### 5.7. Immunologic Response

FIP is classically characterized as an immune-mediated disease, based on early observations of the circulation of complement and immunoglobulins, including as immune complexes [131]. Components of type III and type IV immune reactions have been described [132]. Vasculitis and vasculitis-like lesions are suggested to play a role in systemic complications of COVID-19 that cannot be explained by direct organ infection, such as microthrombosis in the brain, kidneys, spleen, and liver [133]. One report of type III hypersensitivity was identified in the COVID-19 literature [134]; however, immune complexes do not appear to play an important role in the pathology of COVID-19. The mechanism of viral clearance and the inflammatory effects of the immune response are important areas of study for both FIP and COVID-19. Previous work investigating SARS-CoV has demonstrated the necessity of CD4+ T cells for viral clearance [135,136]. T cell depletion has been a recognized consequence of FCoV and has been observed to be associated with more severe cases of COVID-19 [137,138,139]. Additionally, both regulatory T cells and NK cells decrease in FIP disease across blood, mesenteric lymph nodes, and spleen [140]. High levels of IL-6 have previously been demonstrated in FIP ascites [50], and, likewise, elevated IL-6 levels appear associated with disease severity and outcome in COVID-19 patients [141]. Cytokine storm, characterized by the over-expression of inflammatory cytokines, has been implicated in the pathogenesis of both infections. In FIP, this pathology has been linked to the activation of monocytes and macrophages, while in COVID-19, the link to macrophages and monocytes is less clear [142]. In considering the balance between cell-mediated immunity and humoral immunity, early reports indicated an association with strong humoral immunity resulting in FIP [143]. However, in COVID-19 patients, humoral immunity may play a more beneficial role [144], especially given the potential clinical benefits of convalescent plasma/serum [145].

Antibody-dependent enhancement (ADE), the process by which viral–antibody complexes enhance infection, was of particular concern during the SARS-CoV-2 vaccine development process. FIPV has been shown to exhibit ADE in the presence of anti-FIPV antibodies [146]. This enhancement of infection appears to be specific to serotype, with passive immunization of cats against type I or type II FIPV resulting in ADE only after challenging with the same serotype for which immunization was performed [147]. As a result, ADE has been a significant challenge toward the development of FIP vaccines. In human coronavirus diseases, ADE is yet to be fully understood. In SARS-CoV, higher concentrations of anti-spike antibodies were found to have a greater neutralizing effect, whereas more dilute concentrations were suggested to contribute to ADE in vitro [148]. In SARS-CoV-2, ADE was observed in monocyte lineages but was not associated with upregulation of proinflammatory cytokines [149]. Modeling of spike protein sequences identified possible mechanisms for ADE, involving interaction with Fc receptors on monocytes and mast cells [150]. Should ADE play a role in SARS-CoV-2, the most probable mechanism would be through excessive activation of the immune cascade through Fc-mediated activation of innate immune cells [151,152]. At this time, there is not abundant evidence pointing to ADE with SARS-CoV-2 pathogenesis, and further investigation is needed to evaluate the true scope of risk.

## 6. Molecular Similarities between the FCoV and SARS-CoV-2 Spike Proteins

The viral spike protein is a main driver of tissue and cellular tropism and binds the cellular receptor [153]. It is now well established that SARS-CoV-2 binds the angiotensin converting enzyme-2 (ACE-2) as a primary receptor, a feature shared with SARS-CoV. Other binding partners also exist for SARS-CoV-2, including heparan sulfate as a nonspecific attachment and neuropilin-1 (NRP-1), which may account for tropism of the virus for the olfactory and central nervous system [154,155]. In contrast, most alphacoronaviruses, including type II FCoV, utilize aminopeptidases (APNs) for viral entry [9,153,156]. The receptor for type I FCoV remains to be elucidated. The spike protein also mediates membrane fusion, which is activated by an intricate process controlled by host cell proteases [153]. While type I FCoV possesses two protease cleavage activation sites, designated S1/S2 and S2′, FCoV type II only possesses a single cleavage activation site (S2′) [10]. In comparison, SARS-CoV-2 is similar to FCoV-1 (and currently unique for SARS-related viruses) in that there are two identified cleavage sites (S1/S2 and S2′), with the former, the furin cleavage site or FCS, thought to be a significant factor in pandemic spread [157,158,159]. In both cases, the presence of the S1/S2 cleavage sites sets FCoV-1 and SARS-CoV-2 apart from their close family members. The importance of the cleavage activation site appears to link directly to the proteases necessary for viral infection and thus, to an additional component of tissue tropism. In type I FCoV, the transition from FECV to macrophage-tropic FIPV was first shown with amino acid substitutions at the S1/S2 cleavage site on FIP-confirmed pathology samples, which were predicted to downregulate proteolytic priming by furin-like proteases prior to S2′-mediated fusion activation [72,160,161]. In SARS-CoV-2, TMPRSS-2 or other related trypsin-like proteases are the main activator of fusion and entry at S2′ [162] (Table 1), with furin-like proteases priming the spike and S1/S2 [163] and notably shown to be rapidly downregulated upon adaption to Vero E6 cells in culture and possibly also in extrapulmonary human tissues [164]. Thus, there appear to be notable similarities in host cell adaptation between the two viruses.

## 7. Prevention and Treatment: From Social Distancing towards Vaccines

To date, the role of population/public health measures has been a main driver of mitigating the spread of both FCoV and SARS-CoV-2 [3,31,165,166]. In that regard, many social distancing measures have been implemented for affected populations, including stay at home orders, shuttering of nonessential businesses, and limits on public gatherings [167]. Though not termed social distancing, similar methods have been frequently implemented or recommended in feline populations [3]. Dreschler et al. summarize the methods that have been recommended in feline populations, particularly in multi-cat environments, including reducing the number of cats per room, frequent cage cleaning, and grouping cats by shedding and/or serology status [168]. Dreschler states that quarantine of FCoV/FIPV exposed cats to limit the spread of FCoV within the population is neither efficient nor advantageous due to the likelihood of widespread FCoV infection in multi-cat environments as well as the months it takes to develop (and uncertainty in developing) FIP. In contrast, quarantine of SARS-CoV-2 exposed persons has the potential to reduce the spread of disease and death [169]. Regardless of the extent of grouping or separation, careful consideration must be taken into account across both cats and humans with respect to the social difficulties posed by separation. With cats, particularly in the context of early weaning from their queens, special care must be taken in the weaning process to ensure adequate socialization of the kittens. Similarly, with COVID-19, the quarantine and/or isolation process can be mentally burdensome for individuals. Careful cost–benefit analysis must be frequently undertaken to compare the public health benefits of quarantine and isolation with the negative mental toll on persons subject to prevent unnecessary/inefficient quarantine. When necessary, rationale as well as support should be provided to improve wellbeing [170].

While an FIP vaccine is commercially available (Primucell), benefits of FIP vaccination remain low. Primucell is an intranasal vaccine that uses an attenuated serotype 2 FIPV isolate (FIPV-DF2), administered in a two-dose course 3 to 4 weeks apart to cats at least 16 weeks of age [171]. In a placebo-controlled experimental study of 138 cats, vaccinated cats did not show a significantly decreased incidence of FIP compared to controls across the study’s twelve-month observation period. Adjusting for FCoV titers, cats with lower antibody titers (100 or lower) at time of first vaccination compared to those with higher titers (400 or more), had significantly decreased incidence of FIP [172]. However, given the high prevalence of FCoV, especially in multi-cat environments, attempting to mitigate the incidence of FIP through vaccination of FCoV-naïve cats at least 16 weeks of age may be unfeasible given the high potential for FCoV infection in the 16 weeks before vaccine eligibility. Consequently, the American Animal Hospital Association and the American Association of Feline Practitioners does not recommend vaccination against FIP [173].

ADE remains the key concern with FIP vaccines. Several studies have attempted to reduce the incidence of FIP in experimentally infected cats with recombinant and other experimental vaccines, but ADE has repeatedly been suggested. In one placebo-controlled study where purebred British Shorthair cats and specific-pathogen-free (SPF) Domestic Shorthairs were vaccinated with one of two recombinant type 2 FIPV (FIPV-DF2) vaccines, both vaccine candidates showed significantly diminished to no protection against the FIPV challenge in non-SPF cats—with most non-SPF animals showing ADE [174]. In a separate study, the immunization of kittens with vaccinia virus recombined with the spike glycoprotein gene of FIPV significantly reduced survival time after the FIPV challenge compared to kittens immunized to wildtype vaccinia virus. Importantly, low levels of neutralizing antibodies were observed in the FIPV-spike immunized group [175]. The concern of ADE after FIPV immunization remains a difficult challenge in the prevention of FIP.

COVID-19 vaccines, in contrast to FIP vaccination efforts, have played a more prominent role in mitigating the spread of infection. Several vaccine types have been manufactured and demonstrated safety and efficacy in preventing symptomatic infection, severe disease, and death from COVID-19—including but not limited to mRNA vaccines (Pfizer/BioNTech and Moderna), viral vector vaccines (Janssen, AstraZeneca), and inactivated virus vaccines (Bharat Biotech, Sinovac) [176,177,178,179,180,181]. The former two vaccine platforms use the SARS-CoV-2 spike glycoprotein as the immunogen, while the inactivated virus vaccines have the potential to elicit an immune response to other viral components in addition to the spike glycoprotein. Despite the favorable safety profile of COVID-19 vaccines, adverse events after vaccination have occurred, some in an antibody-mediated fashion analogous to the concern of ADE with FIP vaccines. Thrombosis has been a documented concern particularly in the AstraZeneca as well as Janssen vaccines. While the precise mechanisms are under study, the current understanding is where an inflammatory response results in increased levels of platelet-activating antibodies, which bind to platelet factor 4 and result in a hypercoagulable state [182,183]. Unlike the greater incidence of ADE in experimental FIP vaccines, the occurrence of thrombotic events after COVID-19 vaccine administration is low [184].

Beyond the primary endpoints of vaccine studies, which were centered on the prevention of symptomatic infection, severe disease, and death from COVID-19, many of the phase 3 vaccine trials did not engage in surveillance to assess the degree of prevention of asymptomatic infection. Favorable efficacy against asymptomatic infection is important from a public health perspective, especially given the potential for asymptomatic individuals to transmit COVID-19 and that routine surveillance testing is resource-consuming and difficult to coordinate on a large scale [22]. Important contributions toward this area are real-world studies that examine vaccine effectiveness, which point to the decreased risk of infection with SARS-CoV-2 as well as a diminished viral load in vaccine “breakthrough” infections [185,186,187,188]. Such evidence supports the use of SARS-CoV-2 vaccines as a protective measure not only against severe COVID-19, but also as a critical asset in managing incidence of disease.

## 8. Clinical Care and Therapeutic Options

In 1963, when the first clinical cases of FIP were described (prior to knowing the viral etiology), it was noted that antibiotic therapy was frequently attempted, but obviously yielded no benefit [189]. Since this first report, and without an effective vaccine, numerous therapies have been attempted in cats presenting with FIP. Ribavirin, a nucleoside analog, previously provided promising results against FCoV when studied in vitro [190], yet when administered to cats as an experimental treatment, resulted in worse outcomes in some instances [191]. Similarly, in the early part of the COVID-19 pandemic, ribavirin had been utilized at several doses and in combination with additional drugs [192], and a study protocol had been proposed for investigating the benefits in human patients [193]. However, a different direct-acting antiviral (DAA) (remdesivir), a nucleoside analog that acts as a chain terminator and with less toxicity concerns compared to ribavirin, rapidly rose to prominence in treating hospitalized COVID-19 patients, being used in an injectable form. Despite initial enthusiasm, remdesivir has not proven to be effective in such patients in robust clinical trials; however, several reports have demonstrated the clinical benefit of the related nucleoside analog GS-441524 in treating cats with FIP, including effusive, noneffusive, and neurologic forms of the disease [194,195,196,197]. At the time of writing, investigations into the efficacy of remdesivir in treating FIP are being conducted in Australia and the United Kingdom. Interestingly, remdesivir is the pro-drug form of GS-441524 [195]. More recently, two orally-available DAAs have entered clinical trials for COVID-19 and are currently awaiting FDA approval; molnupiravir (MK-4482/EIDD-2801) a modified form of ribavirin, and Paxlovid (a protease inhibitor, PF-07321332, in combination with ritonavir, which improves PF-07321332 half-life) targeting the vial main protease (Mpro). Notably the active ingredient of Paxlovid is related to GC-376 and was previously shown to be effective in a clinical study of FIP [196]. It will be very interesting to follow the course of development, FDA approval, and use of these DAAs in relation to the respective diseases caused by SARS-CoV-2 and FCoV.

Given the inflammatory nature of both FIP and COVID-19, therapy is frequently targeted at controlling the immune response. Though glucocorticoids are frequently given to cats with FIP in an attempt to mitigate the inflammatory sides of the disease, the clinical benefit is negligible [198]. The use of corticosteroids in COVID-19 patients appears not to be without consideration, with some studies showing negative profiles [199]. However, there may be benefits of their administration in severe COVID-19 cases through an observed reduction in mortality [200,201]. Cyclosporine, an immunosuppressive drug often used to prevent organ rejection in transplant patients and the treatment of some autoimmune diseases, has been investigated in both FIP and SARS-CoV-2. An in vitro study of cyclosporine A (CsA) utilizing a type II FCoV virus has shown a decrease in viral replication [202], while treatment of a 14-year-old cat with CsA, following unsuccessful IFN treatment, resulted in clinical improvement, reduction in viral load, and survival time over 260 days [203]. While no controlled trials currently exist in regards to the use of CsA in treating COVID-19 patients, potential mechanisms of action have been suggested in addition to questions regarding safety [204,205,206]. Additionally, the cyclosporine A analogue, Alisporivir, has shown in vitro effects on viral replication [207], similar to evidence demonstrating that replication of other coronaviruses is hindered by the blocking of cyclophilin A [208].

Across both FIP and COVID-19, numerous antibiotics have been prescribed but not for their antimicrobial properties, rather for anti-inflammatory effects [198]. Doxycycline, for example, may have helped with providing prolonged survival in a cat with FIP [209]. Whether doxycycline would exhibit benefit to COVID-19 patients remains unknown at the present, but it has been suggested as a possible component for disease management [210].

Interferons have also been investigated in controlling FIP without clear association in clinical improvement [211]. In human COVID-19 patients, a combination therapy of interferon-β-1b with lopinavir, ritonavir, and ribavirin, compared to just lopinavir and ritonavir was associated with decreased length of viral shedding and improved clinical outcomes in mild to moderate cases [212].

Monoclonal antibodies targeting components of the immune response carry the potential to downregulate inflammatory cytokines. In a small study of cats experimentally infected with FIPV-1146, anti-TNF-α demonstrated benefits for disease management [213]. Tocilizumab, an IL-6 monoclonal antibody, has been administered to COVID-19 patients [214]. More research in regard to Tocilizumab is required, given the disparate clinical outcomes reported [215,216].

The translation of knowledge between species will inevitably create an impact for both cats and humans, and even other species. Though many compounds are effective when studied in vitro, in vivo use can result in different outcomes, including toxicities. Additionally, because a compound may show promise in one species does not mean that the same effect will be observed in other species, especially when comparing similar, but distinct, viruses and virus-induced diseases.

## 9. MIS-C and PASC

In April 2020, the United Kingdom’s National Health Service published an alert of increased incidence of a multisystem inflammatory syndrome in children—many of whom tested positive for COVID-19 [217]. As the pandemic progressed, studies from other countries examining this inflammatory condition have provided more detail toward a clinical understanding of what is now referred to as MIS-C, a rare presentation of COVID-19 in pediatric patients. MIS-C involves multiple organ systems. Cardiovascular dysregulation in MIS-C is often observed in the form of ventricular dysfunction, pericardial effusion, and coronary artery aneurysms [218,219]. Gastrointestinal signs mimic appendicitis and include abdominal pain, vomiting, and diarrhea. Terminal ileitis is a common finding on imaging [220]. Many patients also experience neurocognitive signs including headache and confusion. More severe neurologic complications, including encephalopathy and stroke, are less common [218,221].

One area of significant clinical overlap between FIP and COVID-19 is the rare inflammatory presentation of a SARS-CoV-2 infection—multisystem inflammatory syndrome in children (MIS-C). MIS-C is observed in pediatric populations, similar to how FIP commonly affects young cats [43]. In similar fashion to FIP, MIS-C has a systemic presentation involving multiple organ systems—including but not limited to gastrointestinal, cardiovascular, and hematologic abnormalities [222]. As in the presentation of the wet form of FIP, both pleural effusions and ascites also appear in MIS-C [223]. Both syndromes also demonstrate overlap in the vascular pathology. FIP exhibits a granulomatous vasculitis which shares overlap with the Kawasaki-like vascular syndrome observed in MIS-C [224]. MIS-C has been suggested to be a post-infectious disorder related to prior a SARS-CoV-2 infection [223,225]. FIP, too, has a delayed onset after initial FCoV exposure and only occurs in a small subset of cases. While cats with FIP can still shed FCoV in their feces, mutations associated with the biotype switch from FECV to FIPV are not believed to be transmissible—supporting a degree in similarity of the limited infectious extent of both FIP and MIS-C.

More recently, the condition of post-acute COVID-19 sequelae (PASC) has been defined to include memory loss, gastrointestinal distress, fatigue, anosmia, shortage of breath, etc. and is more commonly referred to as “long-COVID”. Along with MIS-C, PASC is a highly active area of investigation that has been summarized by others [226], and together they provide an excellent starting point for the use of feline medicine as a model for coronavirus-induced pathogenesis, in what might be unexpected ways [224].

## 10. SARS-CoV-2 Infection of Cats

Cats have become now become widely established as permissive hosts for SARS-CoV-2 infections, in part due to the relative similarities of the human and feline ACE2 receptors. Following reported cases in Hong Kong and Belgium during March 2020, the most notable early natural infection was at the Bronx Zoo in New York City, USA. In April, four tigers and three lions developed mild respiratory signs from their keepers, with SARS-CoV-2 detected by PCR and sequencing [227]. Subsequently, infection of both domestic and non-domestic cats has become relatively common where owners and handlers are SARS-CoV-2 positive. Clinically, SARS-CoV-2 infection in cats has been considered to be mainly asymptomatic, with some animals presenting with mild respiratory signs [228,229,230]. In general, severe respiratory signs do not appear to occur in cats, although severe respiratory distress may in some cases be connected to underlying feline hypertrophic cardiomyopathy (HCM) [94]. An increased incidence of canine and feline myocarditis linked to the surge of the B.1.1.7 (Alpha) variant in the UK was also reported [231]. More studies in this area are clearly warranted, as are possible links between coronavirus infections in cats and multisystem inflammatory syndrome in children (MIS-C), which as noted above is a rare presentation of COVID-19.

Studies in laboratory animals have also been key to understanding a SARS-CoV-2 infection in cats, which are highly susceptible to infection by the oronasal challenge. Mild respiratory signs or asymptomatic infection, viral shedding, cat–cat transmission and the development of a robust neutralizing antibody response have all been confirmed in experimentally challenged cats. Recent studies have shown that long-term immunity exists following re-infection of cats, but that cats may develop long-term sequelae, including persistence of inflammation and other lung lesions [232]. In summary, and as with SARS-CoV in 2003, cats in particular may hold important clues to the pathogenesis and immune responses induced by SARS-CoV-2.

## Figures and Tables

**Table 1:** Summary of SARS-CoV-2 and the two FCoV serotypes. The spike glycoprotein of coronaviruses, mediated by proteolytic cleavage, is the main driver of cellular receptor binding and membrane fusion. The taxonomic classification, host receptor, and S1/S2 and S2′ proteolytic cleavage site amino acid sequences are summarized below.

Virus	Group	Receptor	Consensus S1/S2 Sequence in Circulating Viruses	Consensus S2′ Sequence in Circulating Viruses
SARS-CoV-2	Betacoronavirus	ACE2	SPRRAR|S (*SHRRAR|S and SRRRAR|S)	SKPSKR|S
FCoV-1	Alphacoronavirus (“clade A”)	unknown	SRRSRR|S (in FECV; mutated in FIPV)	KR|S
FCoV-2	Alphacoronavirus (“clade B”)	APN	not present	YRKR|S

*, Replaced in Common Variants.
